# Detection of mare parturition through balanced multi-scale feature fusion based on improved Libra RCNN

**DOI:** 10.1371/journal.pone.0318498

**Published:** 2025-03-04

**Authors:** Buyu Wang, Weijun Duan, Jian Zhao, Dongyi Bai

**Affiliations:** 1 College of Computer and Information Engineering, Inner Mongolia Agricultural University, Hohhot, Inner Mongolia, China; 2 Key Laboratory of Smart Animal Husbandry at Universities of Inner Mongolia Autonomous Region, Inner Mongolia Agricultural University, Inner Mongolia, China; 3 College of Animal Science, Inner Mongolia Agricultural University, Hohhot, Inner Mongolia, China; University of Agriculture Faisalabad, PAKISTAN

## Abstract

Once a mare experiences parturition abnormalities, the outcome between a live foal and a stillborn can change rapidly. Automated detection of mare parturition and timely human intervention is crucial to reducing risks during mare and foal parturition. This paper addresses the challenges of manual monitoring of parturition in large-scale equine facilities due to the unpredictability of mare parturition timing, proposing an algorithm for detecting mare parturition through a balanced multi-scale feature fusion based on an improved Libra RCNN. Initially, a ResNet101 backbone network incorporating the CBAM attention module was used to enhance parturition feature extraction capability; subsequently, a balanced content-aware feature reassembly feature pyramid, CARAFE-BFP, was employed to mitigate data imbalance effects while enhancing the quality of feature map upsampling; finally, the GRoIE module was utilized to merge CARAFE-BFP’s multi-scale features, improving the model’s perception of multi-scale objectives and minor feature changes. The model achieved a mean average precision of 86.26% in scenarios of imbalanced positive and negative samples of mare parturition data, subtle parturition feature differences, and multi-scale data distribution, with a detection speed of 15.06 images per second and an average recall rate of 98.17%. Moreover, this study employed a statistical method combined with a sliding window mechanism to assess the algorithm’s performance in detecting mare parturition in video stream continuous monitoring scenarios, achieving an accuracy rate of 92.75% for mare parturition detection. The algorithm proposed in this study achieved non-contact, stress-free, intensive, and automated detection of mare parturition, also demonstrating the immense potential of artificial intelligence technology in the field of animal production management.

## Introduction

Parturition in mares is a complex physiological process, often occurring at uncertain times, late at night or early morning. Dystocia in mares is one of the most common abnormalities in parturition, with a prevalence rate of 4.9%[1] in conventional breeding environments, which might be higher in large-scale breeding farms. Traditional detection of mare parturition relies on manual observation, primarily through scheduled inspections by on-site veterinarians. Traditional parturition detection in mares is time-consuming and labor-intensive, but active close observation can also introduce new stress[2]. Moreover, due to time and spatial limitations, achieving real-time, comprehensive coverage is challenging, often resulting in missed detections. Should a detection be missed and an abnormality in parturition occurs, the mare and foal can quickly become critical. Therefore, achieving real-time, accurate, and automated detection of mare parturition is of significant importance for ensuring the safety of mares and foals during birth, improving the efficiency of equine reproduction management, and enhancing the economic benefits of horse farms.

With the equine breeding industry evolving towards intensification and intelligent technologies, determining parturition in mares through manual patrols no longer meets practical production needs. Therefore, the detection of mare parturition has become a critical issue that the equine breeding industry urgently needs to address. Accelerometers are currently being used to detect mare parturition. Hartmann et al. compared the acceleration changes in mares before parturition and found the most significant changes occurred 30-20 minutes before parturition[3]. Various detection tools based on acceleration have also been employed to detect mare parturition. Aoki and colleagues found that data from several hours before birth has great potential for detecting mare parturition[4]. Skin temperature has also been used to detect mare parturition. Müller and others observed that the skin temperature of most mares increases within 90 minutes before parturition, which can be a potential parameter for detecting imminent parturition[5]. Wearable sensors are used to detect mare parturition through acceleration and skin temperature, which requires the installation of wearable electronic devices on the mare before parturition. In practical applications, the use of wearable devices during the parturition period may influence the behavior of the mare, although there is currently no direct research confirming whether it induces a stress response. Moreover, the mare’s frequent lying down and changes in posture before parturition can easily cause the device to fall off or be damaged, significantly affecting detection accuracy. Temperature sensors are easily influenced by environmental factors such as room temperature, water, and dust. Furthermore, wearable devices also face issues such as power consumption, delays in feedback due to long detection windows, and low detection accuracy.

Mares may be subjected to various stressors during parturition, which primarily arise from environmental changes, physical exertion during labor, and social interactions. Environmental stressors, such as changes in the birthing location, surrounding noise, or the approach of unfamiliar individuals, can activate the mare’s sympathetic nervous system, delaying uterine contractions and the progression of parturition, as confirmed in studies across various animal species [6,7]. Additionally, the physical exertion and pain associated with parturition significantly elevate cortisol levels in mares, resulting in noticeable physiological stress responses [8]. At the same time, mares may become more sensitive to potential threats from other horses or humans during parturition, exhibiting stronger social stress reactions [2,7]. These different types of stress not only prolong the duration of labor but may also increase health risks for both the mare and the newborn foal. Therefore, reducing stressors and optimizing the birthing environment have become critical directions in mare parturition management.

In recent years, with the continuous advancement of computer vision technology, vision-based automated detection methods have been widely applied in the detection of animal behavior. Automated detection methods based on vision technology offer advantages such as being non-invasive, easy to deploy, minimal disturbance, capable of achieving 24-hour comprehensive real-time monitoring, and not causing stress or alarm to animals. Among these, object detection algorithms based on deep learning have become a hot topic in current research[9–14]. Currently, the application of object detection algorithms in the field of livestock is concentrated on individual object detection[15–18], behavior analysis[19,20], posture recognition[21–23], and population statistics[24,25] among others, yet there has been no research on the automation of mare parturition detection using deep learning methods such as object detection. The attention module enhances the model’s ability to capture important information by dynamically focusing on key feature regions, thereby significantly improving the accuracy and robustness of object detection. It has been widely applied in deep learning-related algorithms [26–28].

This paper proposes an object detection algorithm named L-MPD, based on Libra R-CNN[29], to achieve stress-free automatic visual detection of mare parturition behavior and to overcome the impact of imbalanced positive and negative samples and indistinct feature differences on visual detection. Based on the Libra R-CNN algorithm, to address issues such as inadequate feature extraction and representation in complex mare parturition scenes during deep neural network training, this paper utilizes the ResNet101 network[30] integrated with the CBAM[31] attention module to enhance feature extraction capabilities. This paper employs a balanced content-aware feature reassembly pyramid, CARAFE-BFP, to capture multi-object, multi-scale parturition fusion features, thereby tackling poor detection accuracy for distant small targets and under varying scales in mare parturition. This paper adopts a RoI feature extraction method that utilizes multi-layer alignment and information interaction[32] to enhance the model’s robustness and address the issue of poor perceptual accuracy for minor changes in complex scenes. The paper verifies the effectiveness of this method by employing a statistical approach combined with a sliding window mechanism, which analyzes the accuracy of mare parturition identification under video stream continuous monitoring scenarios. Overall, the contributions of this paper are as follows:

(1) A novel method for mare parturition detection has been developed, achieving non-contact, stress-free, real-time automated end-to-end monitoring and decision-making while verifying the feasibility of applying deep learning object recognition technology to mare parturition detection.
(2) A target detection algorithm based on Libra R-CNN, named L-MPD, is proposed. This algorithm incorporates technologies such as attention modules, balanced content-aware feature reassembly in the feature pyramid network, and RoI feature extraction methods based on multi-layer alignment and information interaction, enhancing the accuracy and robustness of mare parturition detection.
(3) This study employs a statistical method that integrates a sliding window mechanism to analyze and verify the accuracy of mare parturition identification under video stream continuous monitoring scenarios. The results indicate that the algorithm can effectively detect mare parturition under video stream continuous monitoring scenarios, achieving favorable identification outcomes.

## Materials and methods

### Data acquisition and pre-processing

#### Experimental mares and stables.

The data for this study were collected from a sizeable equine farm in Ordos, Inner Mongolia. This farm is a professional institution engaged in the breeding, reproducing, and cultivating of equines, boasting significant practical value and credibility. The data were gathered from six parturition areas of the farm, including three outdoor parturition areas, each measuring 12  ×  5.4 meters (L  ×  W), accommodating 1 to 4 mares to move freely and complete parturition, and three indoor stables, each spanning 4  ×  3.2 meters (L  ×  W), each capable of accommodating one mare to move freely and complete parturition. All parturition areas are equipped with iron fences 1.2 meters high and feature individual feed troughs and water troughs to satisfy the mares’ needs for foraging and drinking. Mares nearing their expected parturition dates are dynamically moved to the parturition areas and parturition stables.

#### Parturition video collection.

**Fig 1:**
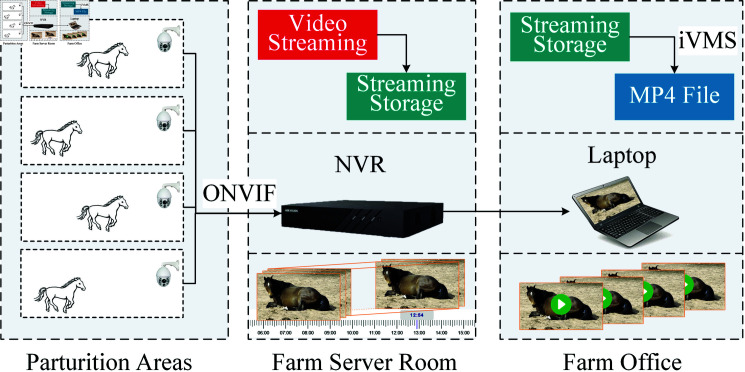
Parturition data collection.

Visible light video images were captured using the spherical camera DS-2DC4223IW-D (Hikvision, Hangzhou, China). The camera’s field of view was 57.6° (wide-angle) to 2.7° (telephoto), with controllable pan-tilt rotation and zoom, a horizontal range of 360°, and a vertical range of -15° to 90°, supporting 23x optical zoom and 16x digital zoom. The captured visible light videos had a resolution of 1920×1080, a bitrate of 4096Kbps, and a frame rate of 25fps and were encoded in H.264. The camera automatically switched to night mode under low-light conditions, capturing video in black and white tones. The night mode automatically activates infrared fill light, with a fill light distance of up to 15 meters. Cameras in the outdoor parturition area were mounted at a corner of the prepartum area, 2.7 meters above the ground, and cameras in the parturition room were fixed to the side walls at a height of 2 meters. All cameras automatically transmitted video data to the mare farm’s NVR DS-7932N-R4 (Hikvision, Hangzhou, China). Within the local area network, laptops installed with iVMS-4200 (V3.5.0.7) were used to control the camera angles and download video data, as illustrated in Fig 1. During non-rainy and non-snowy weather, some mares are transferred to outdoor birthing areas during the day, while all mares are moved back to the indoor stables at night. Therefore, the collected parturition data includes footage from the outdoor birthing area during the day on non-rainy and non-snowy days, as well as footage from the indoor stables during both the day and night. Configuration was done for four experimental parturition area cameras and the control room NVR, adopting a 24-hour continuous video recording scheme. Data collection lasted for two years and three months, and with the assistance of the on-site production director and veterinarians, 152 mare parturition behaviors were collected. All video files were stored in mp4 format, with the filenames based on the recording time.

### Dataset construction

#### Data preprocessing.

The mare parturition process has three stages: early uterine contractions, fetal delivery, and placental expulsion. This paper’s research is confined to the visual identification of the fetal delivery stage, starting from when the elevated white fetal sac becomes visible at the mare’s posterior to the point where the foal’s body is completely separated from the mare. The visible images of this process are defined as mare parturition (MP), while images where this process is not visible are defined as mare non-parturition (MNP). In this paper, one parturition process of the data sample is defined as the period from 15 minutes before the fetal sac becomes apparent to the completion of parturition.

Excluding unusable files for reasons like camera soiling, 146 video files were obtained. To avoid overfitting issues caused by high similarity between consecutive or adjacent frames in the same video and to acquire effective image data, the video files were processed as follows:

(1) From these, 66 longer video files were selected for extracting images to be used as training data, and the remaining 80 shorter videos were reserved for subsequent testing.
(2) Due to the high similarity between adjacent frames, high-quality images were extracted from the videos using FFMPEG (V3.3.9) (with the quality parameter set to 1 for the highest quality output), saving one frame out of every ten as an image file.
(3) All the image files output from the previous step were compared for similarity using the SSIM algorithm[33], retaining only one image from those with a similarity greater than 0.76 to minimize the similarity between adjacent images.
(4) A manual selection process was implemented to eliminate anomalous images caused by obstructions, overexposure, or excessive blurring due to camera focus issues.
(5) To minimize training costs while preserving parturition features, this study employed a Python script to resize all image files generated in the previous step from 1920×1080 to 384×216 pixels and saved them as new image files.

After processing, a total of 5680 usable image files were obtained. As data augmentation will be applied to the training set but not to the test set, the augmented training data will be expanded to twice the size of the original dataset. Consequently, the training and test sets will be randomly divided in a 2:1 ratio. The training set thus consists of 3787 image files, while the test set comprises 1893 image files.

#### Data labelling and enhancement.

The open-source image annotation tool Labelme (version v5.1.1, licensed under the MIT Free Software License) was used to annotate all image files, with the final annotation results converted into the COCO dataset format[34]. The specific labeling standards are as follows:

(1) The entire body of the mare is taken as the input for object detection;
(2) The bounding boxes are tightly fitted around the mares;
(3) If multiple mare targets appear within an area, each target must be annotated.

This study employed the Albumentations library to apply random data augmentation to all training set data, incorporating three augmentation methods: random cropping of images (maximum height of 14, minimum height of 8, maximum width of 14, minimum width of 8), brightness and contrast enhancement (brightness limit of 0, contrast limit of 0 to 1), and image blurring (lower limit of 0.1, upper limit of 0.3). After data augmentation, the number of images in the training set expanded to 7574, with 1893 images in the test set. These augmentation methods can simulate issues such as small object occlusion, excessive image exposure, and camera zooming encountered in reality, thereby increasing the richness of the training samples, enhancing the diversity of the images, and improving the model’s generalization ability.

**Fig 2:**
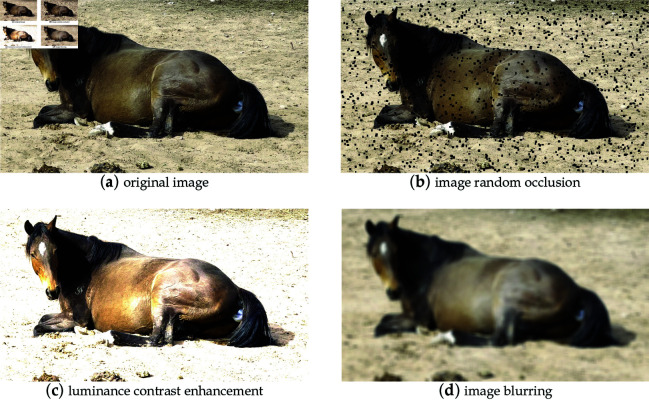
Display of image enhancement effect.

Fig 2 displays a comparison of images before and after data augmentation. It is evident that, following data augmentation, the complexity and diversity of the images have been significantly enhanced, and the effects of occlusion, brightness, contrast, and blurriness within the images contribute to improving the model’s accuracy and robustness, allowing it to better adapt to real-world scenarios and application demands. After data preprocessing, annotation, and augmentation, the final dataset for this study was formed, with the composition of the dataset as shown in Table 1, consisting of 7574 images in the training set and 1893 images in the test set, including a total of 5198 annotations of parturition mares and 11813 annotations of non-parturition mares. As the data collection process yielded far more instances of mares in a non-parturition state than in parturition, despite manual screening during the data preprocessing phase, an imbalance in dataset categories still exists, necessitating the use of specific model training strategies or improvement methods to ensure the model’s capability to identify both categories accurately.

**Table 1:** Equine parturition dataset.

Dataset	Number of original images	Number of enhanced images	Equine parturition	Equine non-parturition	Stable images	Outdoor images	Natural Light images	Artificial Light images
Train	3787	7574	4127	9744	3930	3644	3840	3734
Test	1893	1893	1071	2069	972	921	968	925
Total	5680	9467	5198	11813	4902	4565	4808	4659

#### Establishment of datasets.

After data preprocessing, annotation, and augmentation, the final dataset for this study was formed, as shown in Table 1. The training set consists of 7,574 images, and the test set consists of 1,893 images, with 5,198 annotations for mares during parturition and 11,813 annotations for mares not in parturition. To better reflect typical scenarios in the actual production environment, the dataset includes various environmental conditions such as indoor and outdoor parturition areas, natural light, and artificial lighting, ensuring the diversity of the data. Due to the predominance of non-parturition states over parturition states during data collection, despite manual screening in the data preprocessing stage, there still exists an imbalance in dataset categories that requires specific model training strategies or improvement methods to ensure the model’s ability to recognize both categories accurately.

The process of mare parturition primarily involves two postures: standing and lying down. To assess the model’s generalization ability across different postures, we classified the test set into standing parturition, lateral recumbent parturition, standing non-parturition, and lateral recumbent non-parturition, with the number of images for each type as shown in Table 2.

**Table 2:** Test set labelling details.

Posture	Images of test set
Lateral Recumbent MNP	516
Standing MNP	644
Lateral Recumbent MP	407
Standing MP	326

### Model design

This paper conducts mare parturition detection based on the improved Libra R-CNN algorithm. To address the issue of imbalanced positive and negative samples during the mare parturition process, the Libra R-CNN model integrates three key technological strategies to meet these challenges effectively. Firstly, by employing an IoU-balanced sampling strategy, the impact of sample imbalance during training is significantly reduced, ensuring the quality and efficiency of model training. Secondly, introducing the Balanced Feature Pyramid method has significantly enhanced the model’s ability to detect targets of different scales, especially in identifying subtle feature differences. Lastly, the balanced L1 loss function is applied, effectively reducing the model’s sensitivity to outliers and strengthening its ability to recognize various body positions and states from different angles.

Although the Libra R-CNN algorithm can effectively address the issue of low detection accuracy caused by class imbalance, challenges still remain in the mare parturition detection scenario. These challenges include subtle differences in parturition features, insufficient feature extraction and representation by the algorithm, and poor detection accuracy for small targets at long distances or under varying scales. To further enhance the model’s ability to recognize mare parturition behavior, this study presents targeted improvements to the Libra R-CNN model to enhance its performance. This study investigates the impact of different configurations of attention mechanisms, feature pyramid networks (FPN), and region-of-interest (RoI) extraction modules on parturition detection performance, and designs comprehensive ablation experiments. In terms of attention mechanisms, the study focuses on evaluating the performance of CBAM, SE, and GCA modules. For feature pyramid networks, comparisons are made between FPN, CARAFE-FPN, and CARAFE-BFP. Regarding RoI extraction modules, both RoI and GRoIE modules are compared. Based on Libra R-CNN, this paper proposes an improved mare parturition detection model, L-MPD, which optimizes detection accuracy by deepening the backbone network, integrating attention modules, balancing content-aware feature reconstruction using CARAFE-BFP, and incorporating multi-layer alignment and information interaction-based RoI extraction.

#### The backbone network with integrated attention modules.

In the backbone network design, to enhance the effectiveness and robustness of feature extraction, this study integrates three attention mechanism modules into ResNet101: CBAM (Convolutional Block Attention Module), SE (Squeeze-and-Excitation), and GCA (Global Context Attention). These modules are used to enhance channel attention, spatial attention, and global context modeling, respectively, thereby improving the model’s ability to capture key features in complex scenes. CBAM applies a channel attention module to weigh the importance of features in the feature map, while the spatial attention module focuses on the spatial distribution of the target regions. Woo et al. demonstrated the exceptional performance of the CBAM module in object detection tasks, showing that its lightweight design significantly improves detection accuracy without substantially increasing computational complexity. The SE module, through Squeeze and Excitation operations, recalibrates global features to enhance the expression of salient features. Hu et al. pointed out that the application of the SE module in various convolutional neural networks significantly improves performance in classification and object detection, especially when handling high-resolution images [35]. The GCA module, on the other hand, constructs global features through context-aware mechanisms, enhancing the model’s feature representation in complex backgrounds. Cao et al. introduced the GCA module, which effectively suppresses background noise while improving the quality of the target region’s features, making it especially suitable for object detection tasks in dynamic scenes [36].

In this study, we specifically integrated the attention module into the advanced stages of ResNet101, specifically adding attention module at the end of each residual unit in the third and fourth stages. The purpose of this design is to leverage the feature extraction capability of the attention module for deep optimization and recalibration of high-level features rich in semantic information, thereby effectively enhancing the ability to parse complex semantic features and ultimately improving the model’s capability to capture key visual information related to mare parturition and overall object detection performance.

**Fig 3:**
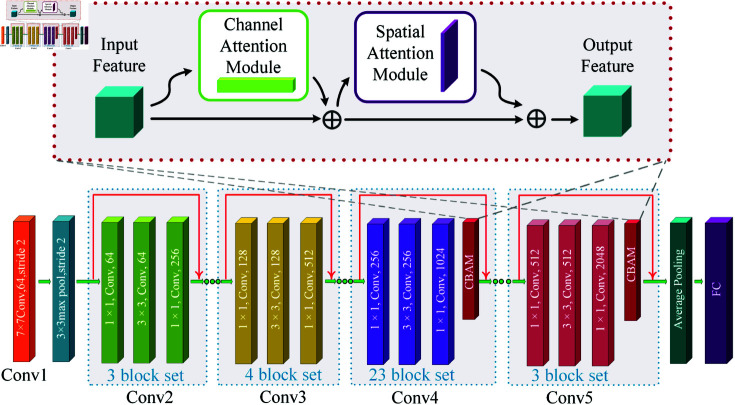
CBAM module architecture. The upper part of the CBAM architecture consists of two components: the channel attention module and the spatial attention module. These modules are applied to the input feature map, and the output is obtained by element-wise multiplication with the input feature map. The lower part shows the specific placement of the CBAM module in ResNet101, where it is added at the end of each residual unit in the third and fourth stages.


As shown in Fig 3, the CBAM module is divided into two parts: the channel and spatial attention modules. The channel attention module first processes the input feature map, then the output feature map is multiplied by the input feature map before being fed into the spatial attention module for processing. After processing by the spatial attention module, a similar operation is performed, and the final output feature map is obtained, with its mathematical expression as Eq (1).

$$\begin{array}{ccc} \begin{array}{c} F^{\prime }=M_{c}(F)⊗F\\ F^{\prime \prime }=M_{S}\left(F^{\prime }\right)⊗F^{\prime } \end{array} & & \end{array}$$ (1)

Where *F* is the input feature map, $M_{c}(F)$ is the feature map of the output of the channel attention module, $F^{\prime }$ is the input of the spatial attention module, $M_{S}\left(F^{\prime }\right)$ is the feature map of the output of the spatial attention module, $F^{\prime \prime }$ is the final feature map of the output of the CBAM module, and  ⊗  denotes element-by-element multiplication.

The SE attention module (Squeeze-and-Excitation Networks) aims to enhance the feature representation capability by adaptively adjusting the channel-wise feature weights. It also offers good computational efficiency, making it suitable for multi-scenario adaptability requirements. In this study, the SE attention module is introduced for ablation comparison. The design of the SE attention module consists of two main steps: the Squeeze operation and the Excitation operation, as shown in Eq (2) and Eq (3).

In the Squeeze operation, the input feature map $X\in {\mathbb{R}}^{C\times H\times W}$, where *C*, *H*, and *W * represent the number of channels, height, and width, respectively, the feature map is first compressed using global average pooling. This operation aggregates spatial information across all locations in each channel, resulting in a channel-wise weight vector $z\in {\mathbb{R}}^{C}$.


$$\begin{array}{ccc} z_{c}=\frac{1}{H\times W}\overset{H}{\underset{i=1}{\sum }}\overset{W}{\underset{j=1}{\sum }}X_{c,i,j},\;c\in \{1,\ldots ,C\}x & & \end{array}$$ (2)

Where $z_{c}$ represents the global feature representation of the *c*-th channel.

In the Excitation opWeration, the compressed feature **z** undergoes a nonlinear transformation through two fully connected layers, generating the channel weights $s\in {\mathbb{R}}^{C}$, and recalibrating the features of each channel.


$$\begin{array}{ccc} s=\sigma (W_{2}\delta (W_{1}z)), & & \end{array}$$ (3)

Where $W_{1}\in {\mathbb{R}}^{C\times C∕r}$, and $W_{2}\in {\mathbb{R}}^{C∕r\times C}$ are the weight matrices, *r* is the scaling factor, usually set to 16; *δ* ( ⋅ )  is the ReLU activation function, and *σ* ( ⋅ )  is the Sigmoid function.

Finally, the channel weight **s** is applied to each channel of the input feature map **X**, with its mathematical expression as Eq (4).


$$\begin{array}{ccc} {\hat{X}}_{c}=s_{c}\cdot X_{c},\;c\in \{1,\ldots ,C\}. & & \end{array}$$ (4)

The SE module effectively enhances significant features and suppresses redundant information, demonstrating good performance in multiple tasks.

The GCA (Global Context Attention) module enhances the feature representation of the target region through global context modeling while suppressing background noise. Its core lies in the computation of contextual information and the adaptive adjustment of weights, as described in Eq (5) and Eq (6).

For context feature computation, given the input feature map $X\in {\mathbb{R}}^{C\times H\times W}$, where *C*, *H*, and *W * represent the number of channels, height, and width respectively, the global context representation $g\in {\mathbb{R}}^{C}$ is calculated by performing a weighted sum over the spatial dimensions.


$$\begin{array}{ccc} g_{c}=\overset{H}{\underset{i=1}{\sum }}\overset{W}{\underset{j=1}{\sum }}p_{i,j}\cdot X_{c,i,j}, & & \end{array}$$ (5)

Where pi,j= exp ⁡ (Xc,i,j)∑ ⁡k=1H∑l=1W exp ⁡ (Xc,k,l) is the normalized weight that indicates the importance of the feature at position  ( *i* , *j* ) .

In context feature enhancement, the global context representation **g** is processed by two fully connected layers to generate the channel weights $w\in {\mathbb{R}}^{C}$.


$$\begin{array}{ccc} w=\sigma (W_{2}\delta (W_{1}g)), & & \end{array}$$ (6)

Where $W_{1}\in {\mathbb{R}}^{C\times C∕r}$ and $W_{2}\in {\mathbb{R}}^{C∕r\times C}$ are the weight matrices, with *r* being the scale ratio (typically set to 16); *δ* ( ⋅ )  is the ReLU activation function, and *σ* ( ⋅ )  is the Sigmoid function.

Finally, the context weight **w** is applied to each channel of the input feature map **X**. with its mathematical expression as Eq (7).


$$\begin{array}{ccc} {\hat{X}}_{c}=w_{c}\cdot X_{c},\;c\in \{1,\ldots ,C\}. & & \end{array}$$ (7)

The GCA module’s approach to contextual information modeling is particularly effective for scenes with complex backgrounds and dynamically changing targets, demonstrating significant performance improvements in video object detection tasks.

#### CARAFE-BFP module.

The Feature Pyramid Network (FPN)[37], as an architecture to improve multi-scale object detection performance, achieves effective integration of features across various scales through a top-down pyramid structure, overcoming the limitations of using single-scale feature maps to adapt to targets of varying sizes. To address the potential blurring and information loss during the upsampling process in FPN, CARAFE FPN[38] utilizes a content-aware feature reassembly approach, dynamically adjusting the upsampling kernels to enhance the accuracy of upsampling and the quality of feature maps. The Balanced Feature Pyramid (BFP) aims to resolve the issue of quality imbalance among features of different scales in FPN. An optimized feature fusion mechanism achieves more balanced information transfer between layers, enhancing detection capabilities for targets of various sizes.

**Fig 4:**
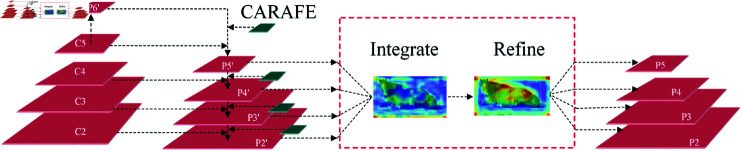
CARAFE-BFP Module. The input feature maps come from the outputs of the FPN, namely P2’, P3’, P4’, and P5’. These feature maps are processed through the Integrate and Refine operations to generate new feature maps P2, P3, P4, and P5, which are used for subsequent object detection tasks.

This paper introduces the CARAFE operator on top of BFP to construct the CARAFE-BFP module based on the Libra R-CNN algorithm, aimed at mitigating the effects of imbalance between positive and negative samples in mare parturition detection while enhancing detection accuracy. As shown in Fig 4, C2 to C5 represents feature maps of different levels obtained from ResNet101, with varying spatial resolutions and semantic richness. By processing these feature maps with the CARAFE module, fusion, and upsampling, we obtain P2’ to P6’. Subsequently, through feature integration (Integrate) and feature refinement (Refine), new feature layers P2P5 are obtained. Integrate merges features P2’ to P5’ from different levels through a specific strategy to integrate multi-scale information. Refine further optimizes and adjusts features based on feature integration, improving features’ representational capability and quality to ensure that the combined features more effectively support subsequent detection tasks.

#### GRoIE region of interest extraction module.

In object detection algorithms, extracting Regions of Interest (RoI) is crucial for locating and identifying targets within images. To enhance the representation of fine-grained features and improve detection performance in multi-scale scenarios, this study introduces a novel RoI extraction module, GRoIE (Generic RoI Extractor). The module aims to optimize the multi-scale feature fusion capability during the feature extraction process, particularly for recognizing mare parturition behavior across different scales in complex scenes.

**Fig 5:**
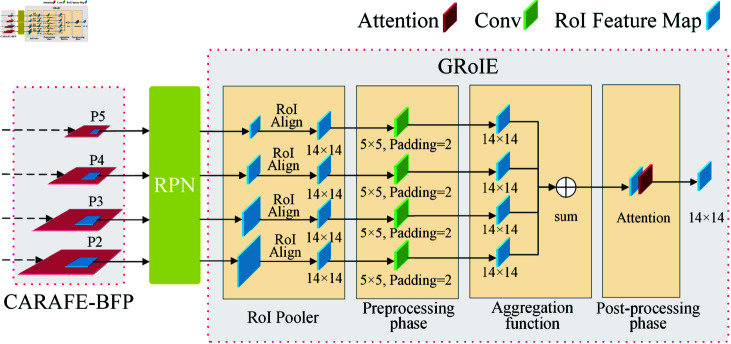
GRoIE Module.

As illustrated in Fig 5, the GRoIE module achieves RoI extraction and enhancement through four steps: RoI Align, Preprocessing phase, Aggregation function, and Post-processing phase. This is important for enhancing object detection tasks in complex scenarios such as mare parturition recognition. Firstly, the RoI Align step extracts features corresponding to the prediction boxes from the feature map through a precise spatial sampling strategy. This study adjusts the output of RoI Align[39] from 7×7 to 14×14, resulting in a higher spatial resolution to ensure the preservation of fine details of mare parturition, laying the foundation for subsequent feature analysis. The Preprocessing phase employs convolution operations to process these features further, enhancing the representation of local details and providing richer information for feature integration. The Aggregation function is responsible for merging features from different sources into a unified feature representation through weighted summation, optimizing information fusion at different scales, and enhancing the model’s adaptability to changes in target states. Lastly, the Post-processing phase introduces an attention mechanism, emphasizing features crucial for parturition state recognition while suppressing background noise, significantly improving the accuracy and robustness of mare parturition recognition.Overall, the GRoIE module achieves efficient multi-scale feature fusion through a staged design. By integrating attention mechanisms, it enhances the selective representation of key features and optimizes the fine-grained processing capability of object detection. This provides comprehensive support for specific target detection in complex scenes and offers a technical guarantee for mare parturition recognition in challenging environments.

#### L-MPD network architecture.

**Fig 6:**
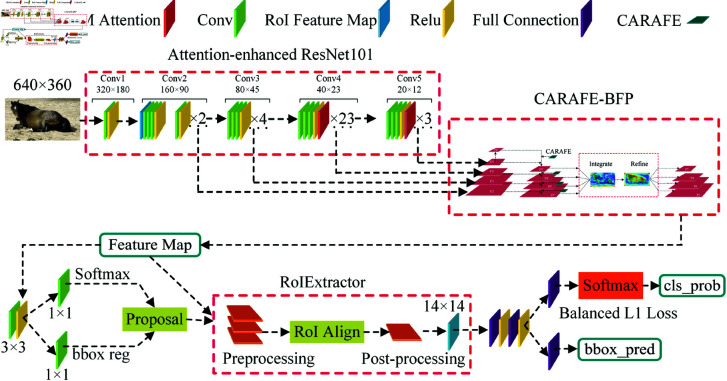
L-MPD network architecture.

This study introduces an L-MPD network model based on an improved Libra R-CNN for target detection of mare parturition. Fig 6 displays the structure of the L-MPD network, comprising four parts: (1) Backbone network utilizing ResNet101, with CBAM attention modules added in Stages 3 and 4 of ResNet101 to extract image features. (2) Employing CARAFE-BFP for further multi-scale feature extraction. (3) Sending the output of CARAFE-BFP to the Region Proposal Network (RPN) to generate region proposals. (4) Based on an improved RoI Extractor (Generic RoI Extractor), integrating all feature maps input from the CARAFE-BFP module for RoI extraction, allowing the extractor to adapt to targets of varying scales and sizes, and handling RoI feature extraction for multiple regions, using RoIAlign to extract features from each proposal box, ultimately performing classification and bounding box regression for parturition and non-parturition of mares.

The backbone network extracts features from the input images for feature map-based target detection. In this study, ResNet101 is employed as the backbone network to extract deep semantic information, with cross-layer connections added in each residual block to address the vanishing gradient and degradation problems during deep neural network training. Additionally, ResNet101 introduces some optimization strategies, such as the Bottleneck structure and Pre-activation module, to improve model performance and reduce computational cost. Its residual structure can be represented by Eq (8). The ReLU activation function is described by Eq (9), and the batch normalization function[40] by Eq (10).


$$\begin{array}{ccc} x_{l+1}=F\left(x_{l},\left\{W_{i}\right\}\right)+W_{s}x_{l} & & \end{array}$$ (8)

In the Eq (8), $x_{l}$ and $x_{l+1}$ denote the inputs and outputs of the lth layer of the network, respectively; *F* denotes a residual block consisting of several convolutions, an activation function (ReLU), and a batch normalization operation, $W_{i}$ is a learnable parameter in the residual block, and $W_{s}$ denotes a weight in the cross-layer connection. In particular, when the input and output dimensions are different, the dimensionality can be adapted by adding a 1 × 1 convolutional layer. For the ReLU activation function, its formula can be expressed as Eq (9).


$$\begin{array}{ccc} f\left(x\right)=max\left(0,x\right) & & \end{array}$$ (9)

The batch normalization function can be expressed as Eq (10).


$$\begin{array}{ccc} BN(x)=\gamma \frac{x-\mu }{\sqrt{\sigma^{2}+\epsilon }}+\beta & & \end{array}$$ (10)

In the Eq (10), BN(x) denotes the result after batch normalization of the input *x*, *μ* and *σ* denote the mean and variance of the current batch, respectively, *γ* and *β* denote the learnable scaling factor and offset, respectively, and *β* is a minimal number that avoids the denominator to be zero.

Instead of the original FPN network, this paper uses CARAFE-BFP further to enhance the feature extraction capability of the model. CARAFE is a lightweight generalized upsampling operator that uses simple interpolation and reassembles the feature vectors according to their similarity to improve the quality of the feature maps when performing the upsampling operation. Its formula can be expressed as Eq (11).


$$\begin{array}{ccc} y_{i,j}=\frac{1}{C}\underset{m,n}{\sum }x_{m,n}\cdot \alpha_{i,j,m,n}\cdot \text{Conv}\left(\psi_{i,j,m,n}\right) & & \end{array}$$ (11)

Where *x* denotes the input feature map, Conv denotes the convolution operation, and *α* and *ψ* denote the position weights and feature reconstruction vectors, respectively. Specifically, $\alpha_{i,j,m,n}$ denotes the contribution of position  ( *m* , *n* )  to position  ( *i* , *j* ) , which can be calculated by the normalized softmax function.

In the RoI feature extraction part of this paper, RoI is extracted from the feature maps outputted from each layer of the CARAFE FPN network, and RoIAlign is used instead of RoIPool to reduce the target location bias of the target detection results. Because the mare partitioning features are much smaller than the mare’s share in the picture, the RoIAlign result is increased from the original 7  ×  7 to 14  ×  14 to detect the mare partitioning behaviours better. Then, the RoI features in the same region are summed and fused to make the model better adapted to the multi-scale target detection task.

### Evaluation metrics of the model

Detection speed FPS, the mean average precision(mAP, IoU=0.50:0.05:0.95), the mean average precision at IOU equal to 0.5 (mAP50), the mean average precision at IOU equal to 0.75 (mAP75), the mean average precision at IOU equal to 0.9 (mAP90), the mean average precision mAP-MP for mare parturition categorization, and mAP-MNP for mare non-parturition categorization, the average recall rate eight indicators as the evaluation index of the model. FPS is the number of images the model processes per second. The calculation method of the mAP evaluation index is adopted from the evaluation index of the COCO dataset, specifically, the average value of AP (Average Precision) for each increase of 0.05 from 0.50 to 0.95 for IoU, and the AP refers to the area under the curve of precision-recall under different IoUs. mAP can use Eq (12) to calculation.


$$\begin{array}{ccc} mAP=\frac{1}{n_{pos}}\overset{n_{pos}}{\underset{i=1}{\sum }}p_{i}\Delta r_{i} & & \end{array}$$ (12)

Where $n_{p}os$ denotes the number of instances of the class, $p_{i}$ denotes the maximum precision value at recall $\geq r_{i}$, $\Delta r_{i}$ denotes the length of the recall interval $\left[r_{i-1},r_{i}\right]$, and the range of recall is $0\leq r_{0}<r_{1}<\cdots <r_{10}<1$. Since the precision-recall curve usually has multiple inflexion points, interpolation is needed to obtain a smoother curve when calculating the AP value. 11-point interpolation is used in the COCO mAP calculation.

The Recall metric is derived from *TP* and *FN*. *TP* denotes the number of correct samples in the identification result, *FN* denotes the number of false-negative samples in the identification result, and *T* denotes the total sample size. See Eq (13).


$$\begin{array}{ccc} Recall=\frac{TP}{\left(TP+FN\right)} & & \end{array}$$ (13)

The method for calculating AR is as follows: First, calculate the Recall at each IoU threshold, i.e., for a given IoU threshold (0.50:0.95), compute the model’s recognition recall rate for positive samples at that threshold. Then, calculate the mean of these recall rates. See Eq (14).


$$\begin{array}{ccc} AR=\frac{1}{\left|Q\right|}\underset{q\in Q}{\sum }\frac{TP_{q}}{\left(TP_{q}+FN_{q}\right)} & & \end{array}$$ (14)

Herein,  | *Q* |  denotes the size of the query set, *q* is the index of the query, and $TP_{q}$ and *FN_q_* represent the number of true positives and false negatives for the query *q*, respectively. This formula calculates the average recall across all queries.

## Results

### Experimental platform and training method

The experimental platform is a computer with the Linux operating system installed, specifically the Ubuntu 18.04 distribution version. The hardware environment consists of 2 Intel(R) Xeon(R) Gold 6139M CPUs @ 2.30GHz, 128GB of memory, and 8 NVIDIA GeForce RTX 3090 graphics cards. The software environment comprises Python 3.9.7, CUDA 11.7.99, Pytorch 2.0.0, MMCV 1.7.0, and the MMDetection 2.28.2 deep learning framework. The hyperparameters for the training process are listed in Table 3. The optimizer employed was stochastic gradient descent with a base learning rate set to 0.005; this learning strategy employs stochastic gradient descent as the optimizer, with an initial learning rate of 0.005, momentum of 0.9; weight decay of 0.0001; all input images are scaled proportionally to 640×360; due to GPU memory constraints, we set the Batch Size to 192.

**Table 3:** Experimental parameter setting.

Hyperparameters	Value
Optimizer	SGD
Learning rate	0.005 with scheduler
Momentum	0.9
Weight decay	0.0001
Input size	640 × 360
Iteration	12300
Batch Size	192

### Selection of backbone networks

This paper selects six commonly used CNN classification networks for comparison as backbone networks: (1) VGG16[41], (2) ResNet34, (3) ResNet50, (4) ResNet101, (5) ResNeSt50[42], (6) ResNeSt101. To enhance the model’s generalization capability, besides adding the commonly used FPN network as the neck network, all other configurations remain the same as the original networks, with all backbone networks pre-trained on ImageNet. Table 4 presents the evaluation results of the test set of Libra R-CNN using different backbone networks. Here, mAP-MP is the mAP value for the mare parturition category, and mAP-MNP is the mAP value for the mare non-parturition category. Except for the AR metric where ResNet34 and ResNet50 exceed ResNet101, and VGG16 processes the highest number of images per second, all other metrics perform best on ResNet101, ultimately selecting ResNet101 as the backbone network for the algorithm.

**Table 4:** Evaluation results of Libra R-CNN with different backbone networks.

Model	Parame-ters/M	FPS	mAP/%	mAP50/%	mAP75/%	mAP90/%	mAP-MP/%	mAP-MNP/%	AR/%
VGG16	31.94	34.07	21.5	35.7	8.02	5.05	21.72	21.28	83.02
ResNet34	38.39	32	69.24	91.32	79.63	17.19	67.37	71.12	97.92
ResNet50	41.35	31.36	70.69	92.21	81.67	16.4	70.01	71.37	97.8
ResNet101	60.35	24.75	82.02	94.88	90.02	36.27	82.65	81.39	97.69
ResNeSt50	43.28	23.86	33.77	61.8	21.45	3.28	33.27	34.28	95.77
ResNeSt101	63.97	14.52	22.87	39.27	8.28	1.04	22.91	22.83	89.24

### Ablation experiment

A systematic ablation study was conducted to examine the effects of model improvements on mare parturition detection performance. The study evaluated combinations of attention mechanisms (CBAM, SE, ECA), feature pyramid networks (FPN, CARAFE-FPN, CARAFE-BFP), and region of interest extraction modules (RoI and GRoIE), with results detailed in Table 5.

**Table 5:** Ablation experiment.

Model	Attention Module			FPN			RoI		FPS	mAP /%	mAP MP /%	mAP MNP /%	AR /%
	**CB-AM**	**SE**	**ECA**	**FPN**	**CARAFE FPN**	**CARAFE BFP**	**RoI**	**GR-oIE**					
Libra R-CNN with ResNet101				*✓*			*✓*		24.75	82.02	82.65	81.39	97.69
	*✓*			*✓*			*✓*		16.82	82.59	82.65	82.54	97.91
		*✓*		*✓*			*✓*		21.03	82.38	82.65	81.72	97.73
			*✓*	*✓*			*✓*		19.26	82.46	82.75	81.85	97.76
	*✓*				*✓*		*✓*		16.38	83.85	84.27	83.43	97.88
	*✓*					*✓*	*✓*		15.67	85.67	86.36	84.98	97.95
	*✓*					*✓*		*✓*	15.06	86.26	86.94	85.57	98.17

When employing the basic FPN and RoI configuration, the model achieved a mean average precision (mAP) of 82.02%. The detection accuracy for the mare parturition category (mAP MP) was 82.65%, while the mare non-parturition category (mAP MNP) reached 81.39%, with an average recall (AR) of 97.69%, indicating relatively modest overall performance. Introducing the CBAM module resulted in a notable improvement, increasing the mAP to 82.59%, with mAP MNP rising to 82.54%, while the detection accuracy for the mare parturition category remained stable. This highlights the effectiveness of CBAM in extracting both global and local features. In comparison, the SE and ECA modules provided incremental performance gains but fell short of the improvements achieved by CBAM.

Replacing the basic FPN with CARAFE-FPN or CARAFE-BFP led to further significant enhancements. The combination of CBAM and CARAFE-FPN achieved an mAP of 83.85%, while integrating CBAM with the more sophisticated CARAFE-BFP increased the mAP to 85.67%. Detection accuracy for the mare parturition category (mAP MP) improved to 86.36%, and the mare non-parturition category (mAP MNP) reached 84.98%. These results demonstrate the effectiveness of CARAFE modules in reconstructing high-resolution features, capturing fine-grained information more effectively.

**Fig 7:**
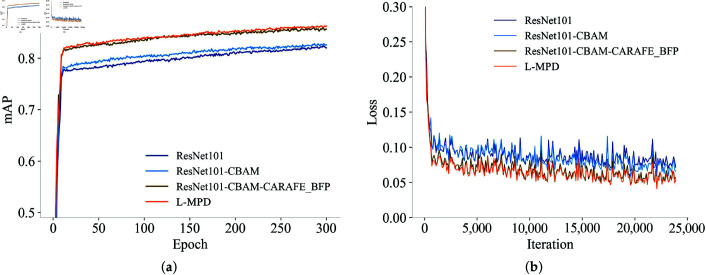
Performance evaluation and training process analysis for key components in ablation study.

Incorporating the GRoIE module achieved optimal performance. The integration of CBAM, CARAFE-BFP, and GRoIE (L-MPD) yielded an mAP of 86.26%, with the detection accuracy for the mare parturition category (mAP MP) improving to 86.94%, and the average recall (AR) increasing to 98.17%. These results validate the pivotal role of the GRoIE module in enhancing feature representation for regions of interest and improving the detection of parturition behaviors under complex conditions. Fig 7 illustrates the variations in mAP and loss for key components of the ablation study throughout the model training process.

### Comparison of standing and lateral recumbent mare parturition identification

Table 6 shows the recognition results of parturition and non-parturition in standing and lateral recumbent states in the test set. It can be observed that the average precision rate of recognition in the standing position is 0.18 percentage points higher than in the lateral recumbent position. The average precision rate for lateral recumbent parturition recognition reached up to 87.7%, with the standing non-parturition average precision rate at 86.7% being higher than the standing parturition rate of 86.03% and the lateral recumbent non-parturition rate of 84.68%.

**Table 6:** Comparison of recognition of standing and lateral recumbent parturition in mare.

Model	mAP/%	mAP50/%	mAP75/%	mAP90/%	mAP-MP/%	mAP-MNP/%	AR/%
All	86.26	95.14	91.89	51.8	86.94	85.57	98.17
Standing	86.37	95.7	91.9	51.45	86.03	86.7	98.98
Lateral Recumbent	86.19	94.21	91.11	52.85	87.7	84.68	95.87

### Comparison with existing target detection algorithms

To test the effectiveness of mare parturition recognition in standing and lateral recumbent states, we compared L-MPD with three object detection algorithms: Faster R-CNN[43] (ResNet101+FPN), FCOS[44] (ResNet101+FPN), and YOLOv3[45] (Darknet53). The evaluation used metrics such as detection speed FPS, average detection accuracy mAP (IoU=0.50:0.05:0.95), detection accuracy mAP50 (IoU=0.5), detection accuracy mAP75 (IoU=0.7), detection accuracy mAP90 (IoU=0.9), detection accuracy mAP MP for the mare parturition category, detection accuracy mAP MNP for the mare non-parturition category, and AR, with YOLOv3 and FCOS being common single-stage detection algorithms, and both Faster R-CNN and L-MPD being two-stage detection algorithms. During the experiment, we trained and tested using a mare parturition dataset and evaluated the performance of each model using the mAP metric. As shown in Table 7, the results indicate that L-MPD overall performs the best on this dataset, with an mAP of 86.26%, significantly outperforming the other three algorithms. The mAP of Faster R-CNN is 81.94%, ranking second, while YOLOv3 and FCOS have mAPs of 75.89% and 79.19%, respectively, showing relatively weaker performance, but FCOS has the best Recall performance at 99.15%. Overall, L-MPD demonstrates good accuracy in mare parturition recognition compared to other algorithms, which better meets practical application needs.

**Table 7:** Different object detection algorithm performance comparison.

Model	FPS	mAP/%	mAP50/%	mAP75/%	mAP90/%	mAP-MP/%	mAP-MNP/%	AR/%
L-MPD	15.06	86.26	95.14	91.89	51.8	86.94	85.57	98.17
Faster R-CNN (ResNet101+FPN)	14.82	81.94	94.29	91.27	37.83	81.96	80.52	98.53
FCOS(ResNet101+FPN)	22.1	79.19	92.36	85.96	36.87	79.76	78.61	99.15
YOLOv3(Darknet53)	26.27	75.89	91.64	84.91	34.19	76.33	74.46	97.92

### Mare parturition detection in continuous monitoring scenarios

**Fig 8:**
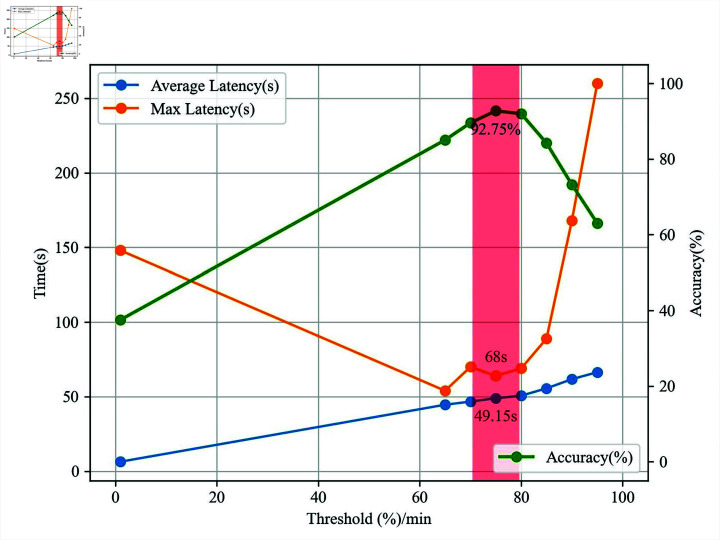
Mare parturition identification under video stream continuous monitoring scenarios.

We employed statistical methods to assess the effectiveness of the L-MPD model in mare parturition detection under video stream continuous monitoring scenarios. We collected video clips of 80 mares from pre-parturition to parturition, with lengths ranging from 4 to 13 minutes. Experts in the field annotated the start time of parturition in the videos, recording the number of seconds from the beginning of the video to the start of parturition. Due to minimal changes in the video frames within a second, to reduce the computational load on the front-end devices as much as possible in actual production, we captured one frame per second from the videos and then sequentially identified the frames using the L-MPD algorithm, taking the highest confidence result as the outcome. We finally evaluated seven methods of parturition recognition: the time of the video corresponding to the first detection of mare parturition was considered the start time of parturition; continuous identification for 60 seconds, with parturition recognized when more than 65%, 70%, 75%, 80%, 85%, 90%, 95% of the images were identified as parturition, and the parturition time was the start time of the continuous 60 seconds. Upon determination of parturition, the program immediately issued a parturition alert to the receiving personnel. The effectiveness of the parturition alerts was assessed from three aspects: accuracy of recognition, average alert delay, and maximum alert delay. The results showed that ’parturition recognized when more than 75% of the images were identified as parturition’ yielded the overall best performance, with an accuracy rate of 92.75% and an average delay of 49.15 seconds, as shown in Fig 8.

## Discussion

This study employed a ten-fold cross-validation technique to assess the model’s mAP and AR to investigate the potential impact of class imbalance on recognition performance. The test dataset was equally divided into ten subsets, with nine subsets combined to calibrate the optimal threshold in each iteration. The tenth subset was used to evaluate model accuracy based on that threshold. This process was repeated ten times, utilizing a different subset as the evaluation set in each iteration to ensure each subset had the opportunity to serve as the evaluation set once. This study derived the model’s comprehensive performance on the overall test data by calculating the weighted average of the accuracy values obtained from these ten evaluations. See Table 8 for detailed data.

Analysis of Table 8 reveals that, throughout the ten-fold cross-validation process, the mean values of mAP and AR for both models remained within a certain range, primarily due to differences in parturition data leading to fluctuations in the same evaluation metric cross-validation results. In terms of mAP analysis, the L-MPD algorithm consistently outperformed Libra R-CNN (ResNet101) in detection performance across validations, mainly because the CBAM attention module and the balanced content-aware feature pyramid effectively extracted key features, a conclusion consistent with the findings of Wangli Hao and others on CBAM. In terms of variance, the mAP and AR variances of our model are 1.53 and 0.67, respectively, indicating more minor fluctuations compared to Libra R-CNN (ResNet101), which suggests more excellent stability of our model.

**Table 8:** Ten-fold cross-validation.

Data Set	Libra R-CNN(ResNet101)		L-MPD	
	**mAP/%**	**AR/%**	**mAP/%**	**AR/%**
The first fold	82.12	98.38	85.04	98.11
The second fold	84.12	95.92	86.27	96.71
The third fold	78.11	96.49	83.79	97.61
The fourth fold	83.39	98.91	87.46	99.42
The fifth fold	79.4	96.48	85.37	98.08
The sixth fold	81.03	97.48	86.45	98.85
The seventh fold	82.4	98.65	86.32	97.32
The eighth fold	83.41	99.65	86.93	99.33
The ninth fold	84.98	96.03	88.47	97.88
The tenth fold	81.27	98.92	86.52	98.4
average value	82.02	97.69	86.26	98.17
variance	4.08	1.71	1.53	0.67
t-test (mAP%)	t = -5.65, p <0.001			
t-test (AR%)	t = -0.98, p = 0.34			

This paper evaluated the performance of different backbone networks on a mare parturition dataset (Table 4). The results indicate that the VGG16 network has limited feature extraction capabilities, performing the worst on the test set, indicative of underfitting. With the ResNet series, as the number of parameters increased, the model’s feature extraction capabilities were more robust, yielding better results. ResNeSt50 and ResNeSt101 performed poorly, only better than the VGG16 network, suggesting that the ResNeSt models captured too much noise in the dataset, leading to model overfitting. Future work will delve into the selection of backbone networks to provide more effective feature extraction algorithms for subsequent target classification and location regression in the algorithm. Through independent samples t-tests, the t-value for mAP was –5.65 with a p-value less than 0.001, further validating the significant improvement of the L-MPD model in mAP. For the AR (Average Recall) metric, the t-value was –0.98 with a p-value of 0.34, indicating that the L-MPD model also maintains good performance in terms of high recall.

**Fig 9:**
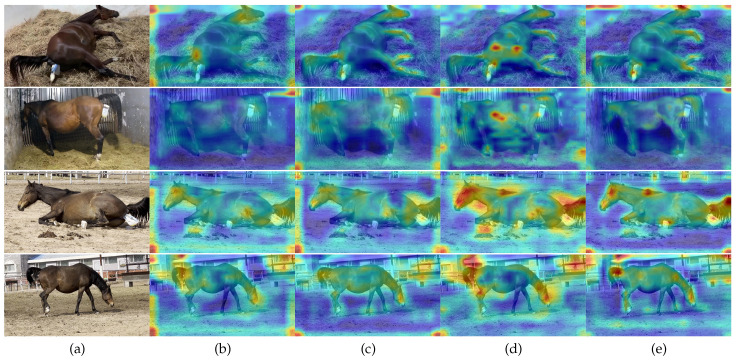
Category activation mapping.

Table 5 shows that through three improvements to the algorithm, we obtained the optimal algorithm L-MPD for this study. From ResNet101 to ResNet101-CBAM, we added an attention mechanism to the backbone network, which slightly improved the mAP (by 0.57%) but reduced the number of images detected per second from 24.75 to 16.82, a decrease of up to 32%. From ResNet101-CBAM to ResNet101-CBAM-CARAFE_BFP, there was a significant increase in mAP (by 3.08%), with a slight decrease in detection speed. From ResNet101-CBAM-CARAFE_BFP to the L-MPD algorithm, there was a slight increase in mAP (by 0.59), with a slight decrease in detection speed. Fig 9 shows the Class Activation Maps (CAM)[46] computed for four mare parturition images using the FeatmapAM (Feature Map Attention Module) algorithm, with Fig 9(a) showing the original image, Fig 9(b) the CAM for ResNet101, Fig 9(c) the CAM for ResNet101-CBAM, Fig 9(d) the CAM for ResNet101-CBAM-CARAFE_BFP, and Fig 9(e) the CAM for L-MPD.

As can be seen from Fig 9, corresponding to the detection results of the algorithm, the introduction of the attention mechanism in the backbone network has a limited improvement on feature extraction, the balance content-aware feature reassembly mechanism introduced by CARAFE-BFP can effectively enhance feature representation, and the generic RoI extractor used by L-MPD further integrates and focuses on the results of CARAFE-BFP. The model ultimately locates the area of interest for parturition detection on the mare’s body and the fetal sac exposed at the tail, demonstrating the effectiveness of the improvements made for mare parturition recognition in this paper, and is consistent with the research findings of Wei Zeng and others regarding CBAM and CARAFE [47].

Through detailed experimental analysis, this study compares the improved Libra R-CNN algorithm (L-MPD) with current mainstream object detection algorithms, including Faster R-CNN, FCOS, and YOLOv3. The performance of each algorithm on mare parturition recognition tasks was evaluated using multiple metrics, including detection speed (FPS), average detection precision (mAP), its variations across different IoU thresholds (mAP50, mAP75, mAP90), specific category mAP, and recall rate. L-MPD demonstrated significant performance improvement in mare parturition recognition compared to existing object detection algorithms. Specifically, L-MPD’s mAP reached 86.26%, ranking first among all compared algorithms, significantly outperforming Faster R-CNN’s 81.94%, FCOS’s 79.19%, and YOLOv3’s 75.89%. This result highlights L-MPD’s superiority in handling complex parturition scenes, especially its detection accuracy at higher IoU thresholds such as mAP75 and mAP90, demonstrating its strong capability in precise recognition. Moreover, L-MPD also maintained a high recall rate (98.17%), proving its effectiveness in reducing missed detections. The outstanding performance of the L-MPD algorithm represents not only a technological breakthrough but also significant practical implications. In a production environment, accurately and timely recognizing mare parturition states is crucial for ensuring the health of both mares and foals. L-MPD’s high mAP and recall rate mean it can reliably detect early signs of parturition, providing a window of time for necessary interventions. Additionally, its high detection speed (FPS) ensures that L-MPD can be applied to real-time monitoring systems, further enhancing its feasibility and effectiveness in practical applications.

**Fig 10:**
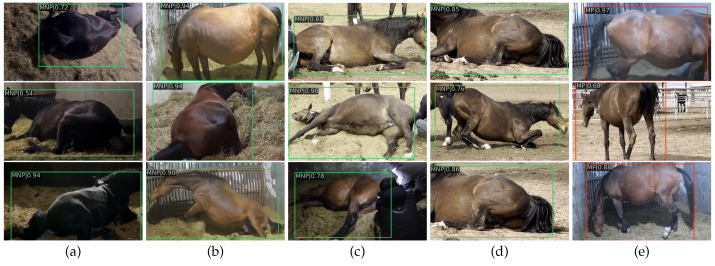
Examples of recognition errors. (a) misidentification due to insufficient lighting, (b) indistinct features due to camera angle and capture range, (c) intrusive interference, (d) tail occlusion, (e) non-parturition mares misidentified as parturition mares due to environmental interference.

Through an in-depth analysis of the misidentification results in this study’s mare parturition detection, the main causes of recognition errors can be summarized into the following five categories, as shown in Fig 10. Fig 10(a) shows misidentification due to dim lighting, where the model struggles to discern the contours of the mare and the details of parturition features, leading to key characteristics of parturition behavior being missed or misinterpreted. This indicates that lighting conditions are an important consideration in environments where mare parturition behavior is being monitored, potentially requiring increased ambient light or illumination in the camera’s direction to improve detection accuracy. In low-light conditions, it is recommended to enable lighting or adjust the camera’s infrared illumination threshold, allowing for automatic switching to infrared mode when illumination is insufficient. The misidentification example in Fig 10(b) reveals the impact of the camera’s angle and capture range on detection accuracy. When the camera is placed in a less-than-ideal position, the parturition features captured are minimal, resulting in the model’s inability to recognize parturition behavior accurately. Therefore, optimizing the installation position and angle of the camera to ensure sufficient coverage of the field of view, along with enhancing the collection and augmentation of training data for small features from multiple angles, is key to improving detection accuracy. Fig 10(c) displays intrusive interference, mainly caused by human intervention in the mare parturition process, which may affect the model’s judgment, leading it to divert attention to non-target objects. Enhancing model training with multi-scenario parturition data to improve the model’s generalization capability may help reduce misidentification, specifically, for scenes where the colors and angles of the clothing worn by delivery personnel interfere with the image, targeted data augmentation should be applied. In Fig 10(d), the mare’s tail obscures most of the key features of parturition behavior, causing the model to fail to recognize parturition behavior correctly. This suggests that during the model training phase, it is essential to introduce more samples containing occlusion scenarios to enhance the model’s feature extraction ability in the presence of occlusions. Additionally, integrating other methods can improve the model’s ability to extract features from challenging and weak-featured samples. Finally, the environmental interference shown in Fig 10(e), leading to non-parturition mares being misidentified as parturition mares, reflects the need for improved model generalization in complex environments. This is due to background noise causing errors in parturition behavior recognition. To address this issue, the model’s generalization ability can be enhanced by incorporating training data from more complex scenarios and introducing background suppression mechanisms.

Although this study has made some progress in improving object detection algorithms, with the final model demonstrating good performance, the research still has some limitations. Firstly, in practical applications, the generalization ability of models poses a significant challenge, particularly regarding their applicability across different environments, breeds, and conditions. This study trains and validates the model using data from a specific equestrian facility, with the dataset primarily encompassing daytime outdoor birthing areas under non-rainy and non-snowy weather conditions, as well as indoor stable birthing scenarios. However, extreme weather conditions, different horse breeds, and environmental variations (such as lighting, temperature, and humidity) have not been taken into account. Variations in different environmental and climatic conditions may lead to changes in the manifestation of birthing behaviors, thereby imposing higher demands on the model’s detection accuracy and robustness.Secondly, although this study employs the Libra R-CNN algorithm that integrates CBAM attention modules and CARAFE-BFP modules to enhance detection performance, these improvements also increase the model’s computational complexity, which may affect the practicality of real-time parturition monitoring applications. Lastly, the parturition posture and position significantly impact the recognition of mare parturition in actual production. If the parturition mare is not tail-end or laterally facing the camera, it is challenging to display parturition features, causing difficulties in parturition recognition. Future research could focus on expanding the diversity of datasets, exploring more efficient algorithm improvement methods, and conducting a more in-depth evaluation of the model’s performance under complex and extreme conditions.

## Conclusion

Due to the uncertainty of parturition timing in large-scale horse farms, which complicates manual monitoring, this paper proposes a mare parturition detection algorithm based on an improved Libra RCNN that integrates multi-scale features. This method builds on the Libra R-CNN object detection algorithm, incorporating the CBAM attention module into the backbone network, using CARAFE-BFP to replace the FPN module, and optimizing the region of interest extraction module with GRoIE. Compared to the original Libra R-CNN and other object detection algorithms, the proposed L-MPD algorithm still achieves excellent recognition performance under conditions of imbalance between positive and negative samples of mare parturition, inconspicuous differences in parturition features, and multi-scale distribution of data. The L-MPD algorithm achieves an accuracy of 86.26% on the test set, processes 15.06 images per second, and has an average recall rate of 98.17%. In video stream continuous monitoring scenarios, using a target detection result statistical analysis method with a 60-second time window, it is concluded that “when more than 75% of the images are recognized as parturition, it is deemed as parturition,” resulting in an optimal overall outcome with a mare parturition recognition accuracy of 92.75%. The experimental results demonstrate that the L-MPD method can accurately detect the parturition status of mares in real time. This research provides a technical reference for constructing unmanned automatic monitoring systems for mare parturition.
